# Science for behavioral health systems change: evolving research-policy-public partnerships

**DOI:** 10.3389/fpubh.2024.1359143

**Published:** 2024-03-13

**Authors:** Michael A. Southam-Gerow, Rafaella Sale, Ashley Robinson, Victoria Sanborn, Juliet Wu, Brianna Boggs, Alana Riso, Marrin Scalone, Ashley Sandman

**Affiliations:** Center for Evidence-based Partnerships in Virginia, Department of Psychology, Virginia Commonwealth University, Richmond, VA, United States

**Keywords:** partnerships, implementation science, evidence-based programs, public mental health system, children’s mental health

## Abstract

Potent partnerships among researchers, policymakers, and community members have potential to produce positive changes in communities on a range of topics, including behavioral health. The paper provides a brief illustrative review of such partnerships and then describes the development and evolution of one partnership in particular in Virginia. The origin of the partnership is traced, along with its founding vision, mission, and values. Some of its several projects are described, including (a) needs assessment for implementation of evidence-based programs (EBPs) pursuant to the Family First Prevention Services Act; (b) statewide fidelity monitoring of key EBPs; and (c) projects to synergize state investments in specific EBPs, like multisystemic therapy, functional family therapy, and high fidelity wraparound. The paper concludes with some themes around which the center has evolved to serve the state and its citizens more effectively.

## 1 Introduction

Readers may be familiar with the ancient parable, dating from early Buddhist and Jainist texts, of the discovery of an elephant by a group of people who were blind. Depending on the version of the story, their efforts to describe the creature led to conflict, confusion, and a less than ideal understanding of the situation. If they had found a way to unify their perspective, perhaps they would have been able to perceive the elephant in its totality. The wisdom found in this story applies to many human endeavors. Faced with large and complex problems requiring multiple individuals to act in a coordinated way, we can struggle to work together to achieve common goals. Public policy implementation poses particularly daunting challenges due to the scope of the problems, the complexity of the systems involved, and the variety of expertise needed to guide action. Research-practice-policy partnerships (hereafter, RPPPs) have long been an important tool in the effort to avoid the elephant effect when implementing new public policy, as they aim to leverage the diversity of skills and knowledge present in a community to enhance the chances of stronger outcomes.

Ideally, RPPPs join a variety of individuals, especially those with expertise in scientific methods, those with practical implementation knowledge and abilities, those affected by any proposed policy, and those with political or other power to affect change. RPPPs are common in many areas of policy implementation including, for example, water use, prevention of medical illness, and education (1–3), and they have been shown to predict policy that supports adoption of evidence-based treatment (4). In this paper, we focus on such partnerships in the behavioral health space, an area with many such arrangements, and one that is prone to challenges in policy implementation due to the many intersecting systems involved. We briefly review the history of partnerships in behavioral health before describing the work of the Center for Evidence-based Partnerships in Virginia (CEP-Va). We trace the origins of CEP-Va and describe an early project in the partnership. We conclude by discussing how our approach to partnership has evolved.

The history of connecting behavioral health research findings to clinical practice and policy has been advocated as a means to reduce the long-lamented research-to-practice gap (5, 6). The tenets of academic and public collaboration to help integrate study results and best practices with the complex realities of service delivery have shaped the missions of a number of organizations across the country. An early example of this kind of partnership is the Connecticut Mental Health Center, founded in 1966 as a collaboration between the state and Yale to provide community services, train community-based clinicians, and conduct academic research (7). In 1976, the scope of work grew to include the Consultation Center—a service, research, and training hub. Also in Connecticut, an effort to improve statewide services and manage growing funds led to the formation of the Children’s Fund of Connecticut, now the Child Health and Development Institute, a nonprofit entity. A third entity in Connecticut is the Innovations Institute. Originally founded in 2005 at the University of Maryland and now housed in the University of Connecticut’s School of Social Work, the Innovations Institute aims to build child, youth, and family-serving public systems that respond to evidence as well as individual and cultural needs, including expanding the workforce responsible for delivering services (8).

Similarly, the University of Washington and Washington State Healthcare Authority (HCA) partnered to create the Evidence-Based Practice Institute (EBPI) to promote evidence-based programs (EBPs) in the state. Originally founded in 2007 by a Washington state bill to improve publicly-administered behavioral healthcare for children, the Institute publishes reports on EBPs in the state, provides guidance for how to report EBPs being provided in the state, publishes a quarterly breakdown of provided EBPs funded by Medicaid, and conducts outreach and partnership efforts to connect quality data reporting and improvements in state-funded behavioral healthcare. The Institute is now housed within CoLab for Community & Behavioral Health Policy in the University of Washington School of Medicine’s Department of Psychiatry and Behavioral Sciences.

In Louisiana, another partnership was established in response to the finding that a gross majority of Medicaid providers reported delivery of EBPs but fewer than half were able to endorse any of the structural components shared by EBPs (e.g., training curriculum for staff) (9, 10). Few providers reported using research-supported practices related to quality assurance such as fidelity monitoring or structured supervision for supporting practitioners in direct service. These findings led to the founding of the Center for Evidence to Practice at Louisiana State University in 2017. To achieve the vision of a state with universal access to high-quality behavioral healthcare delivered by a newly trained or retooled workforce, the center helps the state and its service delivery partners select and implement behavioral health interventions supported by evidence, along with working to understand and address challenges to EBP sustainment.

There are many other excellent partnerships between academic and policy-making organizations in the US that space precludes our mentioning [e.g., California, see (11); Hawai’i see (12, 13); New York State OMH, see (14)]. Many have a similar origin story, emerging in response to a crisis, a lawsuit, or other critical events and becoming sustainable due to the commitment of individuals in critical leadership positions. RPPPs in the behavioral health space have diverse structures. Some are located at academic institutions. Others are independent consultation or research entities. Though each tends to promote behavioral health broadly, the foci of these RPPPs understandably differ based on the sources of funding supporting them and the initiatives active in the state. As a result, behavioral health RPPPs share common challenges and have many unique ones as well. The origin story for the Center for Evidence-based Partnerships in Virginia (CEP-Va) is similar to many such RPPPs, and we turn next to that story.

## 2 The center for evidence-based partnerships in Virginia

Like many states, Virginia has long struggled with its behavioral health system, too often landing in the bottom quartile in national rankings despite some notable initiatives, including the enactment of the Children’s Services Act (1993) and the establishment of community services boards (CSBs) that provide regions across the state with a wide range of services (15, 16). In 2017, the state’s behavioral health agency, Department of Behavioral Health and Developmental Services (DBHDS), worked in tandem with Governor McAuliffe’s Administration, the state’s General Assembly, and other stakeholders to initiate a major reform of Virginia’s behavioral health system called the System Transformation Excellence and Performance (17, 18). STEP-VA required the state’s 40 CSBs to provide nine core services to children and adults: (a) same-day access, (b) primary care screenings, (c) outpatient behavioral health services, (d) behavioral health crisis intervention and stabilization services, (e) peer support and family support services, (f) psychiatric rehabilitation services, (g) veterans behavioral services, (h) targeted case management, and (i) care coordination by July 1, 2021 through several implementation phases (19). Prior to this initiative, CSBs were only required to provide emergency and case management services for adults.

Following on the heels of the STEP-VA initiative, the Virginia Department of Medical Assistance Services (DMAS) and DBHDS formed the Behavioral Health Redesign Workgroup, whose aim was to build a blueprint for a new approach to behavioral healthcare in Virginia via Medicaid expansion. The Redesign group included representatives from various organizations across the state, such as provider organizations, CSBs, professional organizations, advocacy organizations such as the National Alliance on Mental Illness (NAMI), managed care organizations (MCOs), and hospital and healthcare organizations, to contribute perspectives and disseminate information back to their organizations (20). A major focus of their work was the integration of evidence-based programs (EBPs) into all levels of Virginia’s service array. Initially called Project BRAVO (Behavioral Health Redesign for Access, Value and Outcomes), the initiative is now called the Behavioral Health Enhancement (21).

In addition to initiatives like STEP-VA and the Behavioral Health Enhancement, the state also embarked on work related to the 2018 passage of the Family First Prevention Services Act (FFPSA). This landmark federal legislation ushered in major changes for child welfare agencies. Relevant to the other behavioral health initiatives in the state, FFPSA emphasized the development and strengthening of strong arrays of EBPs in communities as a means to reduce use of foster care and other out of home placements. As a result of these multiple EBP-related initiatives, state agencies and their leaders worked to create synergy among EBPs. For example, multisystemic therapy (MST) and functional family therapy (FFT) were introduced throughout Virginia in 2016 by the Department of Juvenile Justice (DJJ) in an effort to reduce recidivism. These two EBPs were also selected by the Virginia Department of Social Services (VDSS) to include in their first Family First Prevention Services Act (FFPSA) prevention plan in 2019. As part of the collaboration around FFPSA, leaders organized an approach coordinating work across all three branches of state government to maximize alignment. Another collaboration was between DBHDS and the Office of Children’s Services (OCS), a major funder of family services. These two state agencies have partnered for years to increase access to High Fidelity Wraparound (HFW) throughout Virginia, a service that reduces out of home placements, a notable goal for the state. Together, these various initiatives have brought a clear focus across multiple state agencies on how best to increase access to quality mental health services across different treatment settings and across the continuum of services.

Given the numerous initiatives, state leaders believed that without intention and coordination, the many related projects would be difficult or impossible to sustain past the launch and even more difficult to evaluate. These leaders, across multiple agencies, leveraged their multi-year collaborations to create an early vision for a center of excellence, located at a state university, designed to provide an independent perspective on the state’s efforts. They approached Dr. Michael Southam-Gerow, an expert in implementation science at Virginia Commonwealth University in Richmond, VA, the state’s capital city, and began to develop a scope for a center of excellence. After multiple iterations, the state team reconvened with enthusiasm and synergy to plan for the funding of the center on the heels of record or near-record budgets for behavioral health redesign in late 2019. Thus, the Center for Evidence-based Partnerships in Virginia (CEP-Va) was born.

The naming of the center as CEP-Va was a product of the state agency partners and Dr. Southam-Gerow, the newly-identified center director, considering the purpose they hoped for from the center. The focus on partnership, thus, was intentional from the beginning. CEP-Va’s collaborative notion with state and local entities is what defines the center. In an effort to capture state leaders’ intentions and ensure internal alignment, the director and postdoctoral researcher (now associate director), Dr. Rafaella Sale, established the vision, mission, and values that guide the center through a rigorous exercise.

***Vision**. We believe all people have a right to resources that promote well-being including high-quality behavioral health services within their own communities. To achieve this vision, we engage in and promote relationships as a key mechanism for large-scale change.*

***Mission**. The Center builds partnerships with stakeholders in public and private organizations to leverage collective support and effort for initiatives designed to improve access to behavioral health services in the Commonwealth. Through thoughtful use of evidence, the Center provides scientific input to stakeholders on the performance of the behavioral health system and paths for enhancing workforce capacity. Alongside its partners, the Center co-designs plans to move Virginia toward equitable, accessible, and evidence-informed behavioral health services.*

***Values**. (a) Inclusion. (b) Integrity. (c) Teamwork. (d) Transparency.*

Before CEP-Va could be fully launched, the COVID-19 pandemic hit and the state budget was redistributed to address the intense needs that arose. After 6 months, the planning team reassembled with the same intention of launching the center, though with few funds to move forward. CEP-Va launched in late 2020 through a small block grant via DBHDS as a temporary funding solution. The state governance committee, chartered officially through the Department of Health and Human Resources, was formed in January 2021 to be comprised of representation from all child-serving agencies in the state. The governance committee has grown since its inception as other state agencies were invited and joined, with the ultimate goal enhancing collaboration among every state agency with a stake in behavioral health. The recruitment of state agency representatives has not been without challenges, with some agencies coming to the table more slowly.

Since its initiation under Dr. Southam-Gerow’s leadership, additional projects have been added to CEP-Va’s portfolio, allowing the team to expand. Indeed, the CEP-Va team has needed to evolve many times during its short existence. In the earliest days, CEP-Va operated like many start-up endeavors, with the small team working across all projects and wearing many hats for the team.

After an initial set of hiring in year two, the team re-organized into three loosely organized groups, including an ongoing needs assessment team, a training team, and a data team. In year three, with additional team members joining, more organization was possible. A leadership team emerged and that team created a set of teams with moderately distinct portfolios of projects, including (a) an engagement and consultation team, (b) a technical assistance materials team, (c) a training team, (d) a service coverage and quality assurance team, (e) a quality improvement studies team, (f) a research team, and (g) an admin team. It is notable that the CEP-Va team is multi-disciplinary and includes team members with degrees in psychology, social work, and public health. Although originally structured by the specific deliverables in its state contracts, CEP-Va has been able to evolve into teams that address key goals derived from its mission and vision and inspired by the founding ambitions of the many state leaders who wanted to work with an independent, academic center.

Details on the funding of CEP-Va may be helpful for others in the field. To date, CEP-Va’s funding has been from state agencies and from private industry (health insurance companies), with most of those funds coming from state agencies. Although the funding to date has been adequate to support the deliverables associated with each contract, there remains a challenge to meet some administrative tasks that cross-cut these projects. An important near-term goal for CEP-Va is to secure infrastructure funding to support the growth of the organization. To this point, the university home has not committed funds to CEP-Va, though support does come in the form of space and other resources. We turn now to a brief review of a few of the projects on which CEP-Va has focused.

## 3 CEP-Va’s scope of work

CEP-Va’s portfolio of projects has expanded greatly in the 3 years since its inception. Although the initial projects focused broadly on several state initiatives, a dominant focus of the early years has been on working closely with the Virginia Department of Social Services (VDSS) in its effort to implement system-wide changes related to the FFPSA. Among the many changes pursuant to FFPSA, the law (a) required each state to file a prevention plan outlining the EBPs to be used by the system; (b) provided funding for training in EBPs from the newly created Title IV-E Prevention Services Clearinghouse, and (c) established fidelity monitoring requirements for all EBPs being implemented. FFPSA presented numerous opportunities and challenges for states, leading VDSS to seek out CEP-Va as a partner in its implementation.

An initial FFPSA-related task for CEP-Va was to help VDSS determine how and where to expand and supplement community service arrays across the state using federal dollars newly allocated through FFPSA. A notable challenge for Virginia lies in its being locally (vs. state) administered, one of only nine such states in the US (22). Local administration means that although the state can set guidelines and some policies, individual counties make some key decisions, including funding of services. Thus, in locally administered states, performance of the child welfare system can vary across counties much more than in state-administered states. As such, understanding local practices, policies, and preferences is paramount. To begin addressing the challenges, CEP-Va developed a unique, ethnographic approach to assess and monitor mental health needs and service gaps within and across Virginia’s five regions and 133 localities. More details are provided in Section 4.

CEP-Va has also served two separate but related roles for Virginia: (a) primary coordinator of EBP training funded by FFPSA monies and (b) creator of fidelity monitoring reports for each of the EBPs in Virginia’s Family First prevention plan. We briefly describe each of these in turn.

**Figure 1:**
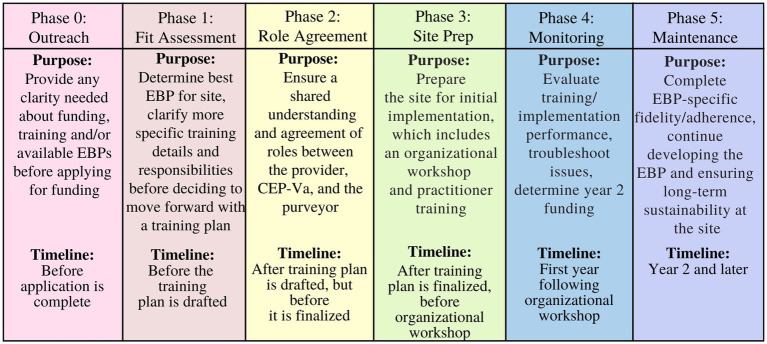
CEP-Va training model.

*Training*. As noted, one result of the FFPSA was a major federal investment in EBP training monies to expand service capacity. VDSS works with CEP-Va to solicit applications from providers to support implementation of EBPs. To accomplish the goal, CEP-Va developed a multiphase training model (see Figure 1) that begins with an *outreach* phase aimed at identifying providers aspiring to expand EBP services and providing information about EBP training available. The next phase, *fit assessment*, focuses on working closely with provider organizations to determine the EBP(s) that best fit the needs of their workforce and the communities they serve. *Role agreement* is the next phase, where CEP-Va develops a training plan that carefully identifies the roles for each player involved in EBP implementation, and outlines the training process and responsibilities in the system for the provider. Once the training plan is complete, the *preparation* phase begins, which includes an organizational workshop wherein the EBP purveyor’s training team meets with the practitioners to be trained, leadership from the provider organization, relevant community partners and referral brokers, state representatives from VDSS and other agencies, and the training support team from CEP-Va. Notably, the preparation phase has evolved in some important ways based on experiences and barriers faced by previous implementation sites. For example, during this phase, CEP-Va works with the provider organization and community workers to expand the service area, when possible, to ensure wider access to the newly established EBP by linking financial support to whole community access. Further, CEP-Va works with the provider organization to build a sound financial plan and ensure sustainability of the service across funding sources. Once the organizational workshop is completed, the formal training process begins, as does the *monitoring* phase. CEP-Va provides scaffolding and support throughout each phase, helping providers reach milestones required for attaining and maintaining site-level certification as an EBP provider in Virginia.

*Fidelity monitoring*. CEP-Va is also responsible for assisting VDSS in meeting its federal fidelity monitoring requirements. The FFPSA mandates fidelity monitoring for all EBPs in a state’s Family First Prevention Plan for which Title IV-E funds were used to pay for services. CEP-Va worked with VDSS and EBP purveyor organizations to develop fidelity models for all EBPs in the state plan and establish data sharing agreements to ensure access to data for the planned reports (see Appendix for glossary of terms). The lack of federal guidance represented an initial challenge. Although the FFPSA stated that fidelity monitoring was required, few details were provided, including what, if any, federal reporting of the resulting data would be.

Another challenge related to fidelity monitoring was that the FFPSA stated that fidelity monitoring was required for services funded by Title IV-E. Virginia, like most states, has struggled to fund evidence-based services through Title IV-E. Because of the state’s early commitment to EBPs, Medicaid established rates for some EBPs and quickly became the major funder of those services. Because FFPSA stipulates that Title IV-E funds may only be used as the payer of last resort, EBPs in Virginia were required to be funded via Medicaid when a family is eligible before Title IV-E funds could be accessed. As a result, developing fidelity reporting for families whose services were solely funded by Title IV-E would only address a small sample of Virginia families. Instead, CEP-Va opted to monitor and report fidelity for EBPs across all funding sources, allowing a more robust and useful fidelity snapshot for the state, consistent with CEP-Va’s overall goals of supporting the state’s system broadly versus an individual agency.

**Figure 2:**
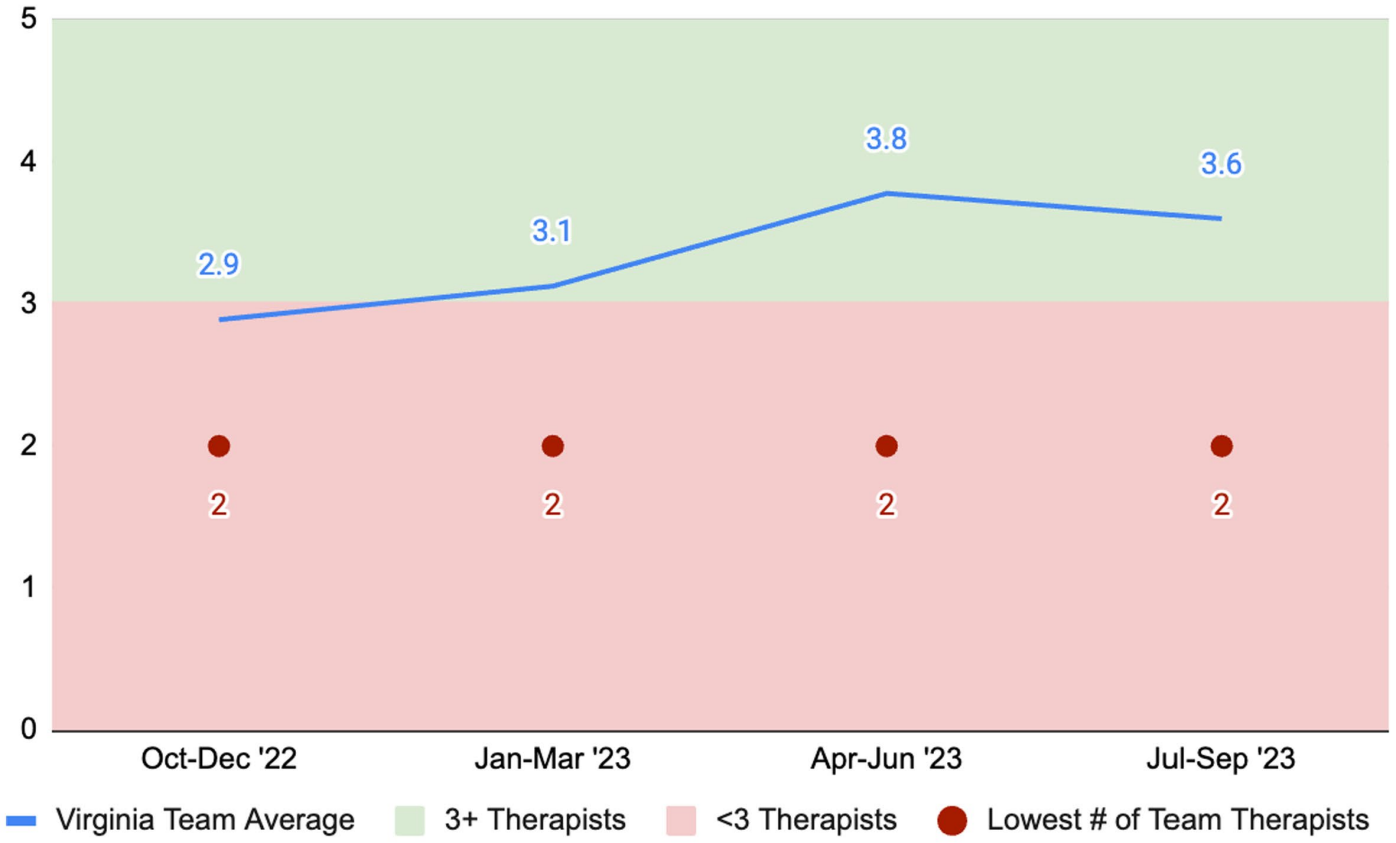
Therapists per team for FFT.

**Figure 3:**
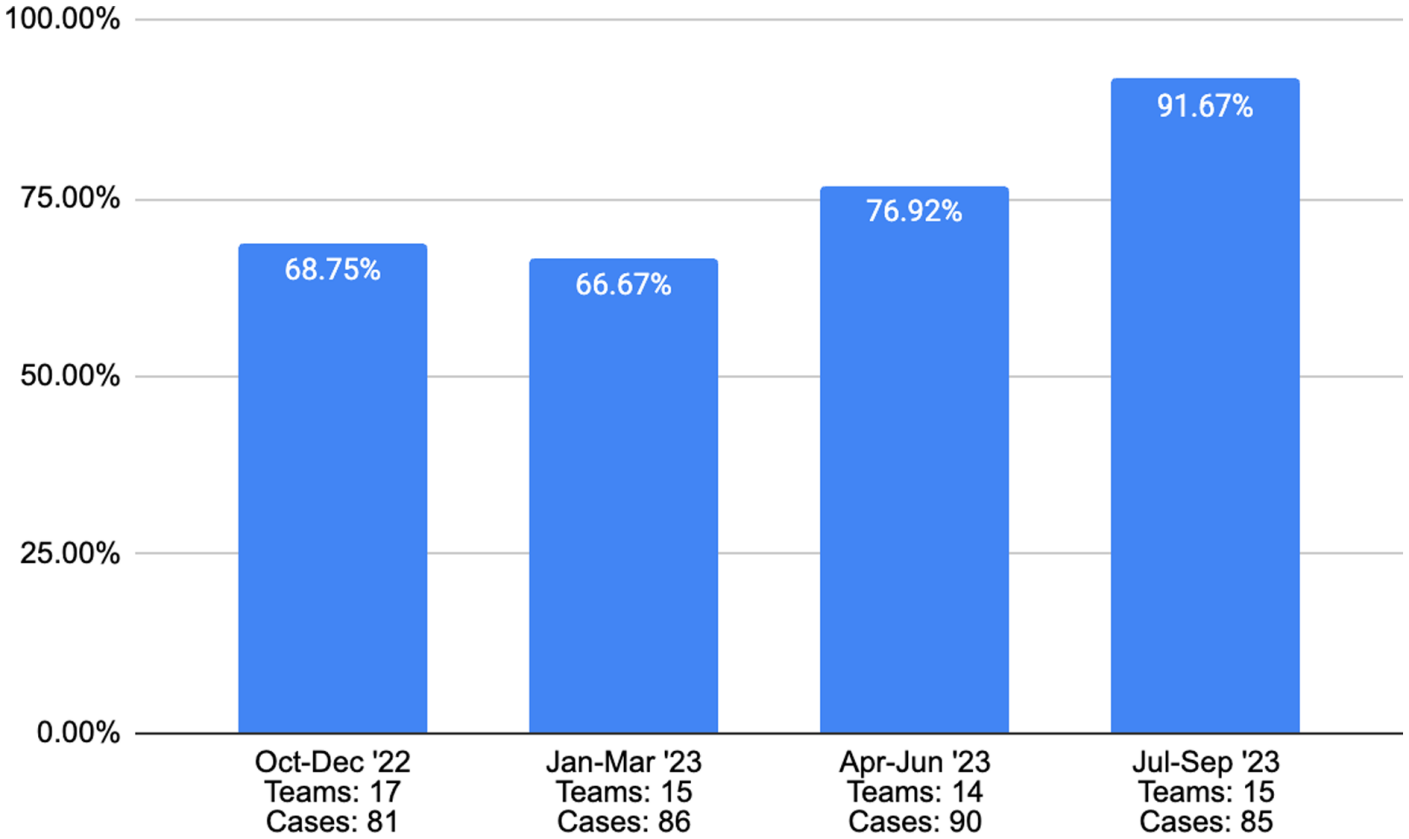
Percent of VA MST teams meeting or exceeding the average fidelity score target.

Figures 2, 3 display samples from recent reports on two of the EBPs in the state’s plan, functional family therapy (FFT) and multisystemic therapy (MST). Both programs have established metrics for multiple fidelity indicators, and our partnership with the purveyors has permitted us to tailor these reports for Virginia. Depicted here are two of the key team fidelity indicators as defined by the purveyor organizations: therapists per team (for FFT) and fidelity scores (for MST). As Figure 2 depicts, Virginia’s teams have struggled to maintain minimum team-size standards. Although the number of therapists per team was above the benchmark for three of the past four quarters, the loss of a single therapist on the average team in half of the quarters would lead to the team being out of compliance. The challenge of maintaining team size is due to many factors captured within CEP-Va needs assessment studies (e.g., workforce shortage, rate changes). In Figure 3, data on fidelity performance for the state’s MST teams are displayed. Fidelity for MST is measured using the Therapist Adherence Measure-Revised (TAM-R), a caregiver-report of the therapist’s fidelity to the principles guiding MST. The purveyor of MST has established that a score of 0.61 or higher on the TAM-R represents an acceptable level of fidelity. Figure 3 thus represents the percentage of teams in Virginia meeting or exceeding that standard. Note that for each quarter, the number of teams and number of cases is reported as added context. Despite the staffing challenges, MST demonstrated strong fidelity scores statewide, with two-thirds or more of teams meeting or exceeding the fidelity standard.

Although FFPSA implementation has been a major driver of CEP-Va activity, another early CEP-Va project was a statewide credentialing database for EBPs. The initial state goal was a single authoritative source to track practitioners trained in at least one of the various EBPs the state was implementing. CEP-Va’s work has yielded two separate online applications. The first, the EBP Registry, is the most straightforward realization of the concept identified by the state. The Registry contains information about each practitioner trained in an EBP, including their workplace, training, and current status in the EBP (or EBPs). Although the Registry relied on a survey to gather some of the data, training status was validated through a CEP-Va developed process to ensure accuracy. The Registry, however, is only searchable by practitioner license number, and thus is only used by the practitioners themselves to confirm and update their own data. The reason for this choice was that provider companies were concerned that a searchable registry of practitioners trained in EBPs would be used as a recruitment tool for companies seeking to hire previously-trained practitioners.

**Figure 4:**
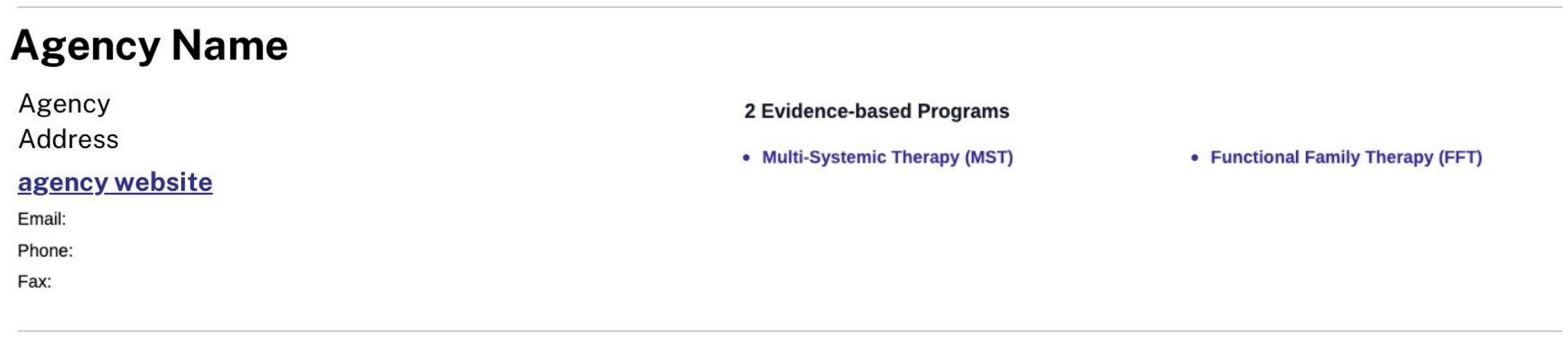
Snapshot of an EBP finder card.

CEP-Va next developed the EBP Finder, a tool that leveraged data from the Registry and was designed for—and in collaboration with—service planners incorporating human-centered design elements [e.g., (23)]. The Finder provides a list of EBPs available by provider company, filterable by EBP, locality, or both. Figure 4 depicts a sample result from using the Finder. In this example, the provider agency listed has been trained and certified to deliver two EBPs at this location.

A final example of CEP-Va’s scope of work is one not spelled out in any of the contracts. Because of the vision, mission, and values of the organization, CEP-Va sought to develop points of communication with its partners, borrowing from community engaged scholarship methods (24–26). As a first approach, CEP-Va began to establish advisory groups with key partners. The first two of these were with provider organizations and managed care organizations (MCOs). The members of the former group were invited after developing a set of selection criteria to ensure representation of the diversity of the state, including demographics of the communities served, state region, size of the provider organization, age of the provider organization, and business model (e.g., for-profit, non-profit). The MCO advisory group is composed of one or more representatives from each of the seven MCOs with Medicaid contracts in Virginia. The focus of the MCO group is sustainment of EBPs, with recent focus being on the adequacy of rates and their provider networks for services demonstrated to reduce residential placement. Future advisory groups will include family members and service planners.

## 4 Examples from CEP-Va’s curriculum vitae

A key initial effort includes the development of an ongoing approach for detecting and monitoring implementation barriers across the state, the Needs Assessment and Gaps Analysis (NAGA) project. NAGA was designed to serve as a springboard for recommended additions to the array of EBPs in the Family First Prevention Plan and to guide the investment of training funds in accordance with regional needs. Detected barriers initiate studies that either end or grow in response to state partner feedback. CEP-Va developed an iterative plan and established some key databases for its use. CEP-Va’s efforts were modeled to be ethnographic (27) and inclusive of the historical and social context to understand how extant systems and their structural linkages influence the various partners in a system, including provider companies, service coordinators, practitioners, and individual families. For its first study, CEP-Va designed and implemented multiple individual projects, leveraging various mixed methods approaches. Some projects focused primarily on qualitative and descriptive data, and others quantitative in nature. Over the past 3 years, the work has resulted in three published reports (28–30). Throughout each iteration of NAGA, CEP-Va maintains a focus on the specific needs of VDSS and the child welfare system, as well as the aims of the state’s many behavioral health partners. In the next few pages, we will describe more examples of the evolution of CEP-Va’s approach to its work.

One constant in CEP-Va’s work has been to leverage extant work in Virginia as a starting point, a process referred to as contextual analysis. To accomplish the goal, CEP-Va has amassed a library of needs assessments and other studies conducted in Virginia over the past decade. These records include documents affiliated with any state or federal government body, legislative proceedings, state, and county-level resource evaluations, publicly-available meeting recordings, and public datasets released by non-profit organizations. Establishing the library has permitted CEP-Va to appreciate how much is already known and understood by policymakers and researchers, to identify knowledge gaps to be filled, and to pinpoint interest convergence among state agencies.

**Figure 5:**
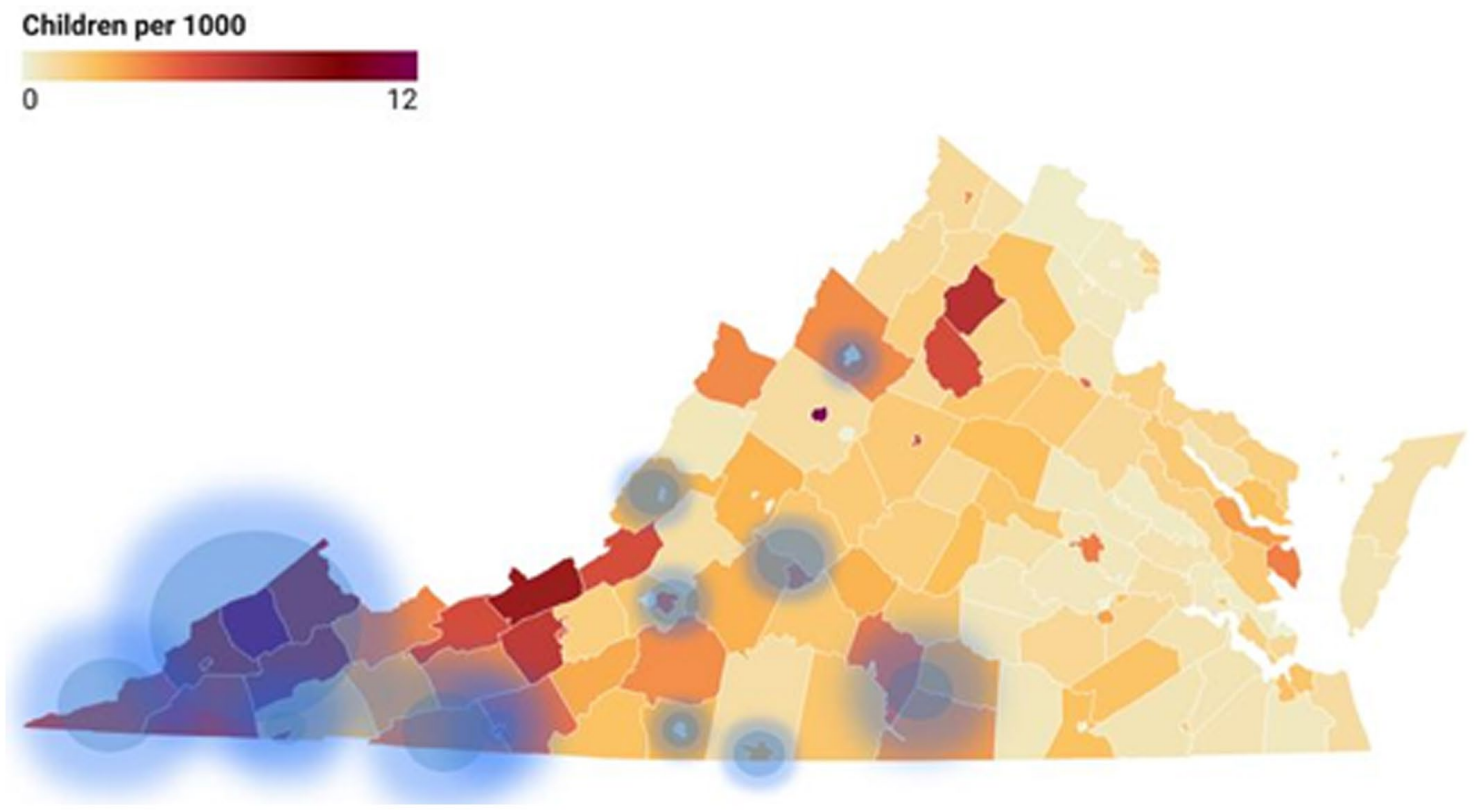
Foster care entry rate FY2021 by locality with families living below poverty level.

One initial result of building this library was a need to focus on specific Community Service Boards (CSBs), the state’s safety-net of publicly-funded community mental health centers. CEP-Va found that almost half (46%) of Virginia’s annual foster care entries in the past 10 years came from the catchment areas of just 13 of the state’s 40 CSBs. Further, through examining the link between poverty and foster care entry, it was found that, out of the state’s 133 localities, the 24 localities with the highest concentration of people living below the poverty line accounted for 33% of children who entered foster care annually. Figure 5 displays the average annual foster care entry rate by locality, with areas of highest poverty concentration highlighted in blue. A key recommendation emerged from these data: support and prioritize the CSBs in these regions, especially by strengthening their deployment of high quality services, including EBPs. A second analysis of data for the Sale et al. report changed the list of priority CSBs somewhat, yet continued to emphasize the importance of supporting CSBs in general.

A second set of findings from CEP-Va studies concerned the challenges facing the workforce in Virginia and across the US [e.g., (31, 32)]. Through a variety of data sources including the library of needs assessments, interviews, and listening fora, a set of obstacles to EBP implementation became clear. In Virginia, as in many states, the licensed workforce accelerated its departure from the public mental health sector during the COVID-19 pandemic. This increasing turnover created struggles for provider agencies to hire and maintain the staff required to implement an EBP. Thus, despite annual training budgets of more than $1 M from VDSS alone, there was limited demand for training. And even when training was initiated, workforce turnover occurred in nearly all cases. As a result, CEP-Va supported the Governor’s Office via their initiative entitled Right Help, Right Now, to examine how restrictions in Virginia regulations or practices exacerbate workforce challenges. One result of this work was a report on how different states in the US deploy unlicensed mental health workers, which indicated opportunities for Virginia to extend the use of an already existing workforce (30).

A related barrier to EBP implementation that arose in CEP-Va’s qualitative work across the state concerns the payment rates for EBPs in Virginia. At the outset, effort was made by the main payors for services to ensure rate alignment, meaning that all funders would use the same rate for the same service. These payors included Medicaid, OCS via Children’s Service Act funds, the Department of Juvenile Justice via diversion grants, and VDSS via Title IV-E funds. Although successful at first, the effort for alignment was derailed and rates for some EBPs began to differ by payor in both reimbursement amount and structure for reporting. Specifically, Medicaid rates for MST and FFT were set for billing in 15 min increments vs. *per diem* like other funders. The result was that provider organizations struggled to use the Medicaid funding in a profitable way, leading to stagnation or decline in the number of MST and FFT teams across the state. It was also a challenge to initiate new EBPs without a specific Medicaid rate because of the requirement to use Medicaid funding if the family was eligible for Medicaid. This constraint led Medicaid providers to seek payment via less-than-optimal reimbursement rates. As one example, brief strategic family therapy (BSFT), an intensive family therapy approach, was paid as an intensive in-home service, a Medicaid service category designed to be delivered by unlicensed practitioners despite the state requirement to staff BSFT with licensed professionals.

To assist the state with these challenges, CEP-Va has taken several steps. First, CEP-Va has used its partnership with multiple state agencies to create regular meetings of key state leaders from the Medicaid agency and other payors to troubleshoot the issues. CEP-Va’s role has been to identify and clarify the challenges and then generate a solution-focused conversation. As noted earlier, CEP-Va has engaged an advisory group composed of representatives from the Medicaid MCOs in Virginia, with an aim to involve them in creating a financial environment conducive to implementation of quality services like EBPs. That advisory group formed a subcommittee specifically focused on the challenges facing MST and FFT teams in the state. One early result of this effort is that one of the MCOs has begun a plan on how to offer enhanced rates for MST and FFT in Virginia. Individual MCOs are permitted to offer rates that exceed the standard Medicaid rate.

Another way CEP-Va has worked to address the rate challenges has been in its work with provider companies seeking EBP training. Through engagement with the companies, CEP-Va evaluates the available workforce at the agency, gaps in service coverage in the region, and the agency’s state and MCO contracts. Doing so aids CEP-Va and the agency determine which EBP will be most suited for their community, workforce, and reimbursement options, ensuring a better chance of financial sustainability despite these rate challenges.

Last, CEP-Va has worked closely with VDSS to identify EBPs for which there is not an existing Medicaid rate and for which such a rate is not planned. One example is High Fidelity Wraparound (HFW), a team-based case coordination approach that requires few licensed workers and can be paid at a higher rate via Title IV-E and state pooled funds alone, because there is not a Medicaid rate, and HFW is not a Medicaid service.

One last example of how CEP-Va’s work has evolved based on the data and its partnerships concerns High-Fidelity Wraparound (HFW). HFW is a community-based, team-based, strengths-focused, collaborative, and individualized process designed to provide a coordinated set of services and support for families with children and youth, from birth to age 21, with complex emotional, behavioral, or mental health needs. HFW is centered on 10 principles, including (a) family voice and choice, (b) cultural competence, and (c) strengths-based. The approach has a rich and developing evidence base supporting its use (33), with evidence suggesting that successful implementation of HFW requires fidelity to the core principles, making training and ongoing coaching a requisite for quality HFW.

HFW has a relatively long history in Virginia and has had the support of multiple state agencies shortly after its introduction. For many years, Virginia had funded training and fidelity monitoring efforts via federal grants, an approach that was successful for some time. Training needs were met by a private provider that founded the Virginia Wraparound Implementation Center (VWIC) while fidelity monitoring was accomplished in several different ways over the years. Although these efforts led to solid expansion of HFW across Virginia, by 2019, there was concern that growth had plateaued or even started to decline. Reliance on federal grants for sustainment of a service incurred several risks. Because funding was frequently uncertain year to year, trainers were difficult to retain, opting to take on full-time employment with more stability. Further, some funding had supported robust fidelity monitoring and others had not. Over time, the provider community did not view fidelity monitoring as a requisite for providing the service. Last, when a grant was not funded, there was a scramble to find funds to tide the operations over for another grant cycle or two. All of these factors left the survival of HFW in a precarious state in Virginia.

CEP-Va was tasked with the development of a sustainment plan for HFW. CEP-Va’s plan of action for the state included several initiatives. First, CEP-Va worked with VDSS to include HFW in a revision of its FFPSA Prevention Plan. Including HFW, considered a Promising Practice in the Title IV-E Clearinghouse, meant that VDSS would have a stake in the service and would be required to conduct an evaluation for the approach. CEP-Va then collaborated with VDSS to write an evaluation plan for HFW in Virginia, one that would focus on the entire state’s HFW implementation rather than the work funded by Title IV-E alone, as was the requirement. CEP-Va would serve as the evaluator, partnering with VWIC to build a feedback system for teams with the training entity.

Next, CEP-Va brokered a deal among state agencies to pool funds and build a funding plan for VWIC. The initial plan included 3 years of funding designed to permit the organization to build out the training and administrative team needed to sustain the work. VWIC’s stability meant that the state now had a consistent training entity for HFW.

Last, CEP-Va worked with leaders from many state agencies to select HFW as the focus for a multi-year transformation zone [e.g., (34, 35)] project in collaboration with the Frank Porter Graham Child Development Institute. The choice meant even more focus on and multi-agency support for HFW in Virginia.

In a brief time, CEP-Va has begun to make an impact on the behavioral health landscape of Virginia. Across projects, the CEP-Va team has held to its founding mission, vision, and values. However, CEP-Va has also evolved in its work, as the team and projects have both expanded. Although much work remains, CEP-Va is a good example of the promise held by sustained engagement of partnerships among policymakers, scientists, practitioners, and other community members.

## 5 Conclusion

Research-practice-policy partnerships (RPPPs) hold great promise to shorten the time frame needed to bring impactful scientific findings to communities and help mitigate social problems. RPPPs are also supremely challenging to maintain, given the various and different forces that influence the behaviors of researchers, practitioners, and policymakers. In our few years in Virginia, several themes have emerged guiding our evolution and we conclude this paper by discussing two of them. Though it is plausible that these themes are Virginia-specific, we hope that they can be helpful for others engaged in this important and difficult work.

The first theme concerns the too oft-overlooked fact that the mental health system operates in a business context. Some early implementation work assumed that the mere existence of EBPs would lead to system-wide change. The thinking was that once providers knew EBPs were available, they would implement them. However, because services were already in place across the system–and that system had adapted to those services–there was (and still remains) a need for significant system disruption to incorporate EBPs.

As discussed earlier, the financing of mental health care represents an enormous test for those who would implement any new service. For EBPs to be implemented, there must be a clear business advantage to them. Some EBPs have understood that necessity from the beginning, and have made their case in policy realms and with financial data. For most EBPs, though, this sort of analysis remains incomplete, posing a significant challenge. The current service system is less expensive than one involving EBPs. EBPs require specialized training and ongoing, paid credentialing. Many also require ongoing fidelity and outcome data collection, in addition to supervisory oversight, including meetings, that reduce productive hours for an employee. In short, EBPs cost more than service as usual to implement. So why, provider companies will rightfully ask, would we change our business practice to a less profitable approach?

Despite the extra cost, EBPs may make good business sense insofar as they reduce future costs, especially with regard to out-of-home placements and other high-cost services. However, specific data are needed to support this hypothesis and for each EBP. Making the moral argument that EBPs are higher quality services is not going to be adequate, given the realities of the US healthcare system. There are likely other cost advantages to EBPs that could be tested in science and then leveraged to support their uptake. For example, if EBPs lead to better outcomes, practitioners may experience improved job satisfaction, tempting them to stay in their current job. Current turnover in mental health positions is costly for provider companies. So, if EBPs lead to better retention, then they save the provider money.

In short, sorting the financing of behavioral health will not be enough alone to lead to a major uptick in access to EBPs. However, failure to sort it will keep things stuck in neutral. Accordingly, we have taken several steps at CEP-Va to ensure that there is a focus on the finance side of our implementation work. First, at the recommendation of an RPPP colleague, our team engaged a national expert on EBP financing to learn more. Further, as mentioned earlier, we created multiple multi-agency meetings to address financing issues at the state level. Primary goals for these meetings are raising awareness of the salience of rates, continuing to align rates across services, and advocating for new rate studies to ensure EBPs are incentivized. Most recently, we have begun to explore working with colleagues in the university’s business school and within Virginia’s provider community to offer business consultation to provider organizations. Although many companies have business backgrounds and/or training in business practices, many provider organizations are run by professionals trained in mental health programs like social work or psychology, with curricula lacking in business training.

A final theme from the early days of CEP-Va has been the centrality of honest relationships. Recall the fable that opened the paper of the individuals who are blind encountering an elephant. The work of RPPPs requires enormous changes to systems involving thousands of individuals, and systems that affect the lives of millions. Such changes require sustained and focused work, effort that requires depth of knowledge and expertise across many different fields. The work also requires human relationships, as it is in those relationships that the solutions are designed, the parts assembled, and the design realized. As all who work in RPPPs know, these solutions can take years to plan and enact. Often, many of the participating partners will have pressures that encourage them to eschew the long-term project in favor of a one-time splashier initiative. As a colleague from another RPPP said in a recent meeting, our task as intermediary organizations can be to remind the state of its own goals and initiatives—to help the state stay on target rather than chase the latest fad. It is easier to accomplish that goal in the context of longstanding partnerships based on transparent communication. At CEP-Va, we have stayed true to our vision, mission, and values. By doing so, we have built strong relationships with numerous partners. And through those relationships, Virginia is beginning to see some positive changes.

## Data availability statement

The original contributions presented in the study are included in the article/supplementary material, further inquiries can be directed to the corresponding author.

## Author contributions

MS-G: Conceptualization, Data curation, Funding acquisition, Methodology, Resources, Supervision, Writing – original draft, Writing – review & editing. RS: Conceptualization, Data curation, Formal analysis, Investigation, Methodology, Supervision, Writing – original draft, Writing – review & editing. AsR: Data curation, Formal analysis, Methodology, Visualization, Writing – original draft, Writing – review & editing. VS: Conceptualization, Project administration, Writing – original draft, Writing – review & editing. JW: Formal analysis, Investigation, Writing – original draft, Writing – review & editing. BB: Formal analysis, Investigation, Writing – original draft, Writing – review & editing. AlR: Investigation, Validation, Writing – original draft, Writing – review & editing. MS: Formal analysis, Investigation, Writing – original draft, Writing – review & editing. AS: Formal analysis, Investigation, Writing – original draft, Writing – review & editing.

## Appendix

Terms defined.

**Table 1:**

Evidence-based practices	specific strategies embedded in interventions that have been proven through highly-controlled research trials to lead to better outcomes, i.e., empirically-supported
Evidence-based program(s)	manualized treatment packages that have been shown to work in research trials when delivered close to exactly the way they were developed
Evidence-based service(s)	a broad umbrella term for which programs exist underneath, referring the all the service components (ex., evidence-based programs, case coordination) that together contribute to a family receiving high-quality care
Feedback system	routine schedule of measuring indicators of quality or impact on outcomes, usually through a digital platform or data dashboard, for the purpose of guiding and informing delivery of a program or practice in real time
Fidelity	the degree to which a program adheres to specific model standards as determined by model developers
Implementation	multi-phasic process of integrating scientific findings into routine practice that emphasizes identification of factors that affect uptake of a novel practice or intervention
Policymakers	state and local governmental employees who are tasked with writing regulations and rules in an effort to apply and abide by federal and state laws
Practitioners	individual therapists, clinicians, counselors delivering services directly to children and/or families in any setting; includes unlicensed clinicians
Progress monitoring	general activity of collecting data for the purpose of assessing any type of movement toward a goal, objective, or desired status
Provider	companies or agencies that deliver behavioral health services; not individual direct service providers such as therapists or clinicians
Purveyor	program developers, trainers, or vetted spokespeople who represent an evidence-based program, and have a clear stake in how the program is delivered
Referral brokers	individuals in a service system such as caseworkers or case management specialists who refer families to behavioral health service providers
Sustainment	the active maintenance of gains or defined outcomes related to an innovation; ultimate goal of implementation
